# Cure Rates After a Single Dose of Radioactive Iodine to Treat Hyperthyroidism: The Fixed-Dose Regimen

**DOI:** 10.7759/cureus.28316

**Published:** 2022-08-23

**Authors:** Nneka M Madu, Catherine Skinner, Samson O Oyibo

**Affiliations:** 1 General Practice, Peterborough City Hospital, Peterborough, GBR; 2 Radiotherapy, Peterborough City Hospital, Peterborough, GBR; 3 Diabetes and Endocrinology, Peterborough City Hospital, Peterborough, GBR

**Keywords:** single dose, graves´disease, overt hypothyroidism, fixed-dose, hyperthyroidism, radioactive iodine, cure rates

## Abstract

Introduction

Radioactive iodine (RAI) has been used to treat hyperthyroidism for more than 70 years. Cure rates after RAI therapy range between 80% and 100%, with some patients requiring two or more doses. There is continued debate over which dosing regimen is optimal. We evaluated our cure rates after giving a single dose of radioactive iodine to treat hyperthyroidism using the fixed-dose regimen as opposed to the calculated-dose regimen.

Materials and methods

We retrospectively reviewed the clinical records of patients who had received their first single dose of RAI between 2016 and 2021. The patients had clinical and biochemical assessments every six weeks until six months post-RAI therapy, then every three months thereafter, if still not cured. Patients were deemed cured if they developed persistent hypothyroidism or euthyroidism after a single dose of RAI. The data included baseline demographics, adverse events, and cure rates after RAI treatment.

Results

One hundred and thirty-eight patients received their first dose of RAI during the study period. Their mean ± standard deviation (SD) age was 56.9 ± 15.3 years, and there were 101 women and 37 men. The median duration of hyperthyroidism was 34 months, and 62% of the cases were affected by Graves’ disease. A majority of patients (90%) were on an antithyroid drug prior to RAI therapy. The median (interquartile range) dose of RAI received by the group was 559 (546-577) megabecquerels (MBq). Four patients (2.9%) reported adverse events shortly after receiving RAI. Our overall cure rate was 87.7% amongst patients who received a single dose of RAI therapy. This number consisted of 96 patients (69.6%) who developed hypothyroidism and 25 patients (18.1%) who remained euthyroid. Our one-year cure rate was 84.1%. Further analysis revealed that women had a greater cure rate than men over the study period (92% vs 75.7%, p = 0.017).

Conclusion

We have evaluated cure rates after a single dose of RAI therapy for the treatment of hyperthyroidism at our center. Our results are comparable to those reported at other centers using a similar dosing regimen.

## Introduction

Hyperthyroidism is an endocrine disorder characterised by an inappropriately high production of thyroid hormone by the thyroid gland. A United Kingdom follow-up community survey of thyroid disease found that the prevalence of hyperthyroidism in women was as high as 2% and that it was 10 times more common in women than in men [1]. A majority of cases are due to Graves’ disease, and the rest are due to either multiple toxic nodules or a solitary toxic nodule within the thyroid gland. Treatment regimens include antithyroid drugs, radioactive iodine, or surgical thyroidectomy [2]. Radioactive iodine (RAI) has been used to treat hyperthyroidism for more than 70 years and is indicated in cases of hyperthyroidism due to Graves’ disease, multi-nodular goitre, and solitary toxic nodules. RAI can be given as first-line or second-line therapy after the failure of antithyroid drugs [3]. This therapy employs the use of iodine-131, which is a beta-emitting radioisotope of iodine. Radioactive iodine is selectively concentrated within the thyroid gland and gradually destroys the thyroid gland over time [4]. Cure rates after RAI therapy range between 80% and 100%, with some patients requiring two or more doses [3].

Debate still exists over the method of establishing the optimal dose of RAI for patients. Some centers use a calculated-dose approach, based on formulas that take into account timed radioactive iodine uptake measurements and thyroid gland volume. Other centers use a fixed-dose regimen, with treatment doses ranging between 300 and 800 megabecquerels (MBq). There is also debate over the use of low-dose RAI versus high-dose RAI [2].

At our center, we use the fixed-dose regimen for the treatment of hyperthyroidism in adults. The aim of this study was to evaluate the cure rates after giving a single dose of radioactive iodine to treat hyperthyroidism at our center.

## Materials and methods

This was a retrospective study. We reviewed clinical records and collected data on all patients who had received their first single dose of RAI between the years 2016 and 2021 at Peterborough City Hospital, United Kingdom. Data included baseline demographics, adverse events, and cure rates after RAI treatment. The approval for this retrospective analysis of clinical and laboratory information with a waiver of consent was obtained from the Department of Research and Development, North West Anglia NHS Foundation Trust.

Hyperthyroidism was diagnosed on the basis of elevated serum free thyroxine (FT4) and/or free triiodothyronine (FT3) values and suppressed thyroid-stimulating hormone (TSH) values. Subclinical hyperthyroidism was diagnosed on the basis of normal serum FT4 and FT3 values with suppressed TSH values. To ascertain whether the hyperthyroidism was due to Graves’ disease or toxic nodules, we used a combination of history, clinical examination, biochemical profile, TSH receptor antibodies (TRAb), and, if necessary, thyroid ultrasound and a radioiodine uptake scan.

Patients on an antithyroid drug (carbimazole or propylthiouracil) were asked to stop these seven days before RAI therapy. Patients with active thyroid eye disease had a prophylactic course of steroid therapy (prednisolone) starting a week before RAI therapy and the dose was tapered over six weeks. On the treatment day, the RAI was swallowed in capsule form. We have two fixed RAI dose prescriptions at our center: a standard dose (550 MBq) and a high dose (800 MBq). The high dose was prescribed to improve the chances of cure in patients who were non-tolerant of antithyroid drugs, non-compliant with taking antithyroid drugs, non-responsive to high-dose antithyroid drug therapy, and patients who had severe cardiac dysfunction, in whom an exacerbation of hyperthyroidism would have been clinically dangerous. The RAI capsules are not manufactured onsite but are delivered on the day of treatment. Therefore, the actual administered dose has always varied within plus or minus five per cent of the originally prescribed dose.

Follow-up data for the year following RAI therapy and throughout the study period were collected. Patients had clinical and biochemical assessments every six weeks up until six months post-RAI therapy, and every three months thereafter if still not cured. The onset of cure was defined as subnormal to normal serum FT4 and FT3 levels and normal to raised TSH levels, indicating the onset of biochemical hypothyroidism (elevated TSH). Thyroid hormone replacement, in the form of levothyroxine, was initiated once laboratory evidence of hypothyroidism was established. Euthyroidism was defined as normal thyroid hormone (TSH, FT4, FT3) levels without the need for thyroid hormone replacement after receiving a single dose of RAI. Therefore, the term “cured” was defined as the onset of persistent hypothyroidism or hypothyroidism requiring thyroid hormone replacement or the onset of persistent euthyroidism after receiving a single dose of RAI therapy. The term “not cured” was defined as the persistence of subclinical hyperthyroidism or hyperthyroidism requiring antithyroid drug therapy, another dose of RAI or surgical thyroidectomy during the study period.

Statistical analysis

Statistical analyses were performed using IBM SPSS Statistics for Windows version 28.0 (IBM Corp., Armonk, N.Y., USA). Whole numbers and percentages were used to present categorical variables. Percentages of the total within each category were used to present cure rates. Mean and standard deviation (SD) were used to summarise parametric continuous variables, while median and range were used to summarise non-parametric continuous variables. Two-tailed Student t-test and Mann-Whitney U test were used to compare baseline parametric and non-parametric variables, respectively. Two-tailed Fisher’s exact test was used to detect differences in cure rates within categories. A p-value of less than 0.05 was considered statistically significant.

## Results

Baseline demographics and clinical characteristics

Over the six-year period, 138 patients received their first dose of RAI for the treatment of hyperthyroidism. The mean ± SD age of the group was 56.9 ± 15.3 years, with a range of 24-89 years. The group comprised 101 women and 37 men. The median duration between the diagnosis of hyperthyroidism and receiving RAI treatment was 34 months. Most of the patients (62%) had a diagnosis of Graves’ disease, while 17.4% were secondary to either a solitary toxic nodule or multiple toxic nodules. A majority of patients (90%) were on an antithyroid drug (carbimazole or propylthiouracil) up until seven days before the RAI treatment. A dose of 550 MBq was prescribed for 88.4% of patients while the 800 MBq dose was prescribed for the remaining 11.6% of patients for reasons stated above. The median (interquartile range) dose of RAI received by the group was 559 (546-577) MBq. Thirteen patients had a documented history of thyroid eye disease, but only six of these had active disease and received prophylactic steroid therapy. Table 1 shows patient baseline demographics and characteristics.

**Table 1:** Baseline demographics and characteristics of patients MBq: megabecquerels, RAI: radioactive iodine, TRAb: TSH receptor antibody

Baseline characteristics	Categories	N (%)
Age (mean ± SD years)		56.9 ± 15.3
Gender	Female	101 (73.2)
	Male	37 (26.8)
Aetiology of hyperthyroidism	Graves’ disease	85 (61.6)
	Solitary toxic nodule	8 (5.8)
	Multiple toxic nodules	16 (11.6)
	Amiodarone-induced	1 (0.7)
	Not specified	28 (20.3)
TRAb result	Positive	62 (44.9)
	Negative	32 (23.2)
	Not done	44 (31.9)
Pre-treatment antithyroid drug	Carbimazole	127 (92.0)
	Propylthiouracil	5 (3.6)
	None	6 (4.4)
Duration of hyperthyroidism (median (range) months)		33.5 (3-372)
Thyroid eye disease		13 (9.4)
Prescribed RAI dose	Standard dose (550 MBq)	122 (88.4)
	High-dose (800 MBq)	16 (11.6)
Actual dose of RAI received	Median (range) MBq	559 (508-862)
Reported adverse events		4 (2.9)

Immediate adverse events

Four patients (2.9%) reported adverse events after receiving RAI treatment. The first patient was a man who developed a severe exacerbation of hyperthyroidism requiring admission to the hospital. The second patient was a woman who developed an exacerbation of pre-existing thyroid eye disease. She admitted that she had not taken her prescribed steroid prophylaxis. The third patient was a man who developed an exacerbation of pre-existing thyroid eye disease despite being on prophylactic steroid therapy. He required high-dose intravenous steroid therapy. The fourth patient was a woman who developed an erythematous, nodular rash over her lower limbs. These skin lesions were radioiodine-induced dermatopathy (iododerma). The lesions resolved spontaneously after three to four months.

Cure rates over the study period

Over the study period, the overall cure rate was 87.7% amongst patients who received a single dose of RAI therapy at our center. This number consisted of 96 patients (69.6%) who developed hypothyroidism and 25 patients (18.1%) who remained euthyroid after a single dose of RAI. Seventeen patients remained uncured; this included seven patients who went on to have a second dose of RAI, two patients who went on to have total thyroidectomy, and eight patients who remained on an antithyroid drug for continued hyperthyroidism. At the one-year post-RAI therapy follow-up point, 84.1% of patients were cured. This consisted of 88 patients (63.8%) who developed hypothyroidism and 28 patients (20.3%) who were euthyroid at one-year post-RAI therapy. The cure rates at the various clinical and biochemical assessment points are shown in Table 2.

**Table 2:** Cure rates for the use of radioactive iodine to treat hyperthyroidism Values are patient whole numbers (percentages) RAI: radioactive iodine

Treatment outcomes	Duration after RAI therapy					
	6 weeks	3 months	4.5 months	6 months	12 months	After 12 months
Hypothyroidism	19 (13.8)	59 (42.8)	82 (59.4)	85 (61.6)	88 (63.8)	96 (69.6)
Euthyroidism	9 (6.5)	17 (12.3)	21 (15.2)	23 (16.7)	28 (20.3)	25 (18.1)
Cured (hypothyroidism or euthyroidism)	28 (20.3)	76 (55.1)	103 (74.6)	108 (78.3)	116 (84.1)	121 (87.7)

Of the 138 patients in our treatment group, 16 patients (11.6%) were prescribed the higher dose (800 MBq) of RAI to increase the chances of achieving a cure from a single dose. Three-quarters of the group had Graves’ disease. The group received a median dose of 830 MBq. Twelve patients (75%) achieved cure (hypothyroidism) after a single dose. No patient became or remained euthyroid. The remaining four patients had continued Graves’ hyperthyroidism: two patients had a second dose of RAI, one patient had a total thyroidectomy and the fourth patient remained on an antithyroid drug. The proportion of patients that became hypothyroid (requiring thyroid replacement) among the group that had the higher dose, was greater than the same in the group that had the standard dose (Table 3). However, this difference did not reach statistical significance (75% vs 68.9%, p = 0.776).

**Table 3:** Treatment outcomes using standard dose versus high-dose radioactive iodine therapy Values are patient whole numbers (percentages) within categories RAI: radioactive iodine

Treatment outcomes	Prescribed dose	
	Standard dose RAI	High-dose RAI
Hypothyroidism	84 (68.9)	12 (75)
Euthyroidism	25 (20.5)	0
Not cured	13 (10.7)	4 (25)

Baseline factors and overall cure rate

Analysis revealed that women had a greater cure rate than men over the study period. This difference was statistically significant (92% vs 75.7%, p = 0.017). There were no statistically significant differences in the baseline age, duration of hyperthyroidism, aetiology of hyperthyroidism, and the dose of RAI received between the “cured” group and the “not cured” group over the study period (see Table 4 and Table 5).

**Table 4:** Baseline categorical variables for the “cured” and “not cured” groups Values are patient whole numbers (percentages) within categories *Solitary toxic nodule and multiple toxic nodules combined

Variables	Categories	Outcome after RAI		P-value
		Cured	Not cured	
Gender	Female	93 (92.1)	8 (7.9)	0.017
	Male	28 (75.7)	9 (24.3)	
Aetiology of hyperthyroidism	Graves’ disease	73 (85.9)	12 (14.1)	0.742
	Nodular thyroid*	21 (87.5)	3 (12.5)	
	Not specified	26 (92.9)	2 (7.1)	
	Amiodarone	1 (100)	0 (0)	

**Table 5:** Baseline continuous variables for the “cured” and “not cured” groups MBq: megabecquerels, RAI: radioactive iodine, IQR: interquartile range

Variable	Outcome after RAI		P-value
	Cured	Not cured	
Age (mean ± SD years)	57.2 ± 15.2	54.8 ± 16.3	0.543
Duration of hyperthyroidism (median (range) months)	35 (3-372)	23 (8-108)	0.321
Dose of RAI (median (IQR) MBq)	559 (546--577)	560 (555-693)	0.189

## Discussion

The dose of RAI used to treat hyperthyroidism varies from center to center, with cure rates (persistent hypothyroidism and euthyroidism) ranging from 70 to 90% [5]. Some centers use a single fixed-dose regimen or several fixed doses, while others use a dose calculation based on the thyroid gland radioiodine uptake and volume-weight estimates. Because of these multiple approaches, the actual RAI dose given to patients can vary between 200 to 800 MBq across centers around the world [5]. There is little evidence that using a calculated-dose regimen has any advantage over a fixed-dose regimen in treating hyperthyroidism; more research is required to reach a consensus [2].

The success of RAI therapy in Graves’ disease strongly depends on the administered dose. Randomised controlled trials have found a success rate of 81% with the 555 MBq dose, and 86% with the 580 MBq dose [6]. The American Thyroid Association recommends a mean RAI dose of 370-555 MBq to render a patient with Graves’ disease, hypothyroid. A higher dose (370-740 MBq) is recommended for patients with toxic nodules. Therefore, the aetiology of hyperthyroidism should be determined if a diagnosis of Graves’ disease is not clinically or immunologically apparent [6]. Patients should stop antithyroid drugs at least two-three days before RAI therapy and can restart antithyroid drugs three-seven days after RAI if there is an increased risk associated with exacerbation of hyperthyroidism [6]. Patients should be followed up within one-two months after RAI therapy with a full thyroid hormone assessment at four-six week intervals for six months, or until the patient develops hypothyroidism [6]. The Royal College of Physicians has made similar recommendations. It also suggested a higher dose (500-800 MBq) for patients with severe co-morbidity such as severe heart failure, malignancy or psychosis, and patients who are intolerant of antithyroid drugs or with thyroid eye disease, to reduce the need for a second dose of RAI [3].

Several studies have attempted to tease out factors that affect the efficacy of RAI [7]. Age, gender, thyroid size, smoking, antithyroid drug pre-treatment, thyroid hormone and autoantibody levels, and applied RAI dose have all been culprits in different studies. Many of these studies are retrospective, lack a control group, have sample selection bias, or the sample size is too small [7]. A single factor that reliably predicts the outcome of RAI therapy remains unidentified.

The overall cure rate after a single dose of radioactive iodine to treat hyperthyroidism at our center was 87.7%. A fifth of these patients remained euthyroid for the rest of the study period. Our cure rate at one year was 84.1%. Our cure rates are similar to those found at other center using a similar dosing regimen [8-10]. We also note that some centers have reported cure rates above 90% using a similar dosing regimen [11,12]. As mentioned previously, our radioactive iodine capsules are supplied to us and the actual dose given to the patient can be below the prescribed dose by 5%. This could have contributed to slightly lower cure rates at our center.

It should be noted that between the second and fourth years after RAI therapy, three of our patients switched from being euthyroid to being hypothyroid, and five patients switched from being thyrotoxic on an antithyroid drug to being hypothyroid. This emphasizes the importance of long-term follow-up for those patients who have not become hypothyroid after receiving RAI.

There were no significant differences in the cure rates among the aetiology groups. The documentation for the aetiology of hyperthyroidism was incomplete (20% with aetiology not specified). We could not find a TRAb result for 32% of our patient group, and some of these patients did not have further thyroid imaging. Although recommendations mention the possible need for a higher RAI dose for nodular thyroids [3], the presence of nodular thyroid disease did not influence RAI dosing at our center.

The only baseline characteristic that was different among the "cured” group and the “not cured” group was gender. Women had a greater cure rate than men. Previous studies have demonstrated conflicting results; females have a greater cure rate in some studies and males have a greater cure rate in others [13]. A large meta-analysis is required to examine this further.

Eleven per cent of our patients were prescribed a higher dose of RAI (800 MBq). These patients had an adverse reaction to antithyroid drugs, no clinical response to antithyroid drugs, were non-compliant with taking their antithyroid drugs or had severe cardiovascular disease that continued or rebound hyperthyroidism would have been clinically dangerous. We aimed to increase the chances of cure after a single dose of RAI, thereby reducing the chance of continued or rebound hyperthyroidism and reducing the need for a second dose of RAI. This was achieved in 75% of patients in this group. However, we were expecting a 90-100% cure rate after such a high dose tailored for this specific group of patients. Although the high-dose RAI did produce hypothyroidism without any euthyroidism, and the proportion of hypothyroidism was greater than that produced by the standard dose, our results did not reach statistical significance as in previous studies [10,14]. This was likely due to small numbers. Large prospective randomised controlled trials are required to examine this further.

Three per cent of our patients reported side effects after receiving RAI therapy. The literature reports transient thyroiditis, exacerbation of hyperthyroidism, and exacerbation of pre-existing thyroid eye disease as the main immediate side effects of RAI therapy [7]. However, the incidence rates of these side effects are not clearly defined. Thyroid eye disease can occur de novo after RAI therapy in 15-33% of patients with Graves’ disease. However, pre-existing thyroid eye disease and smoking are risk factors for the exacerbation of eye disease after RAI therapy [7]. The occurrence of radioactive iodine-induced iododerma in one of our patients was a new phenomenon for us; this particular case was reported [15]. 

Things to improve upon

Firstly, we should aim to determine the aetiology of hyperthyroidism in all our patients. All patients should have a TRAb test, especially in the absence of clinical features of Graves’ disease. Patients with a negative test for TRAb should have thyroid gland imaging (radioiodine uptake scan and, if necessary, an ultrasound scan). It should be pointed out that many of the cases in this report were historical, having had hyperthyroidism for many years before joining our center. Secondly, we need to get our RAI manufacturer-supply-delivery interval on point, such that the administered activity is as close (less than 5% variation) to the prescribed activity as possible.

## Conclusions

We have evaluated cure rates after a single dose of RAI to treat hyperthyroidism, using a fixed-dose regimen at our centre. The data demonstrated that we achieved an overall cure rate of 87% and that women had a greater cure rate when compared to men. Our results are comparable to that reported at other centers using a similar dosing regimen. Large randomized trials and meta-analyses are required to establish the optimum dose of RAI and factors affecting cure rates when treating hyperthyroidism. 
